# Different Inhibitory Interneuron Cell Classes Make Distinct Contributions to Visual Contrast Perception

**DOI:** 10.1523/ENEURO.0337-18.2019

**Published:** 2019-03-11

**Authors:** Jackson J. Cone, Megan D. Scantlen, Mark H. Histed, John H. R. Maunsell

**Affiliations:** 1 Department of Neurobiology, University of Chicago, Chicago, IL 60637; 2 Unit on Neural Computation and Behavior, National Institute of Mental Health, Bethesda, MD 20814

**Keywords:** inhibition, interneurons, mouse, psychophysics, vision, visual contrast

## Abstract

While recent work has revealed how different inhibitory interneurons influence responses of cortical neurons to sensory stimuli, little is known about their distinct contributions to sensory perception. Here, we optogenetically activated different genetically defined interneurons [parvalbumin (PV), somatostatin (SST), vasoactive intestinal peptide (VIP)] in visual cortex (V1) of mice working at threshold in a contrast increment detection task. The visual stimulus was paired with optogenetic stimulation to assess how enhancing V1 inhibitory neuron activity during visual processing altered task performance. PV or SST activation impaired, while VIP stimulation improved, contrast increment detection. The impairment produced by PV or SST activation persisted over several weeks of testing. In contrast, mice learned to reliably detect VIP activation in the absence of any natural visual stimulus. Thus, different inhibitory signals make distinct contributions to visual contrast perception.

## Significance Statement

Inhibitory interneurons are diverse and influence sensory responses through multiple mechanisms. Currently there is little consensus on how different inhibitory signals affect sensory perception. Here, we investigated how genetically defined interneuron subclasses [parvalbumin (PV), somatostatin (SST), and vasoactive intestinal peptide (VIP)] influence visual contrast perception. Mice were trained to work at threshold in a visual contrast detection task and interneuron activity was enhanced using ChannelRhodopsin-2 (ChR2) on a subset of trials. Different interneuron classes influenced perception in distinct ways. PV and SST stimulation impaired, whereas VIP stimulation improved visual contrast perception. With training, mice learned to perceive VIP activation in the absence of natural visual stimulation. These data highlight how different inhibitory signals affect the transformation of sensory-evoked responses into percepts.

## Introduction

In the visual system, inhibitory interneurons contribute to fundamental sensory computations like stimulus selectivity (Priebe and Ferster, 2008; Liu et al., 2011), response normalization (Carandini and Heeger, 2011), and center-surround suppression (Ferster, 1988; Adesnik et al., 2012). This wide-ranging influence depends in part on their genetic, physiologic, and morphologic diversity (Markram et al., 2004). The vast majority of inhibitory interneurons in cerebral cortex belong to one of three genetically defined interneuron classes that express parvalbumin (PV), somatostatin (SST), or vasoactive intestinal peptide (VIP), although each class is likely to include distinct cell types that differ along many dimensions, especially morphologic (Rudy et al., 2011).

Transgenic mice that provide access to PV, SST, or VIP neurons for optogenetic targeting have helped clarify how different sources of inhibition augment stimulus-evoked responses in sensory cortices. PV and SST neurons, which target somata and dendrites, respectively (Rudy et al., 2011; Kubota et al., 2016), suppress visual responses in pyramidal output cells (Atallah et al., 2012; Cottam et al., 2013; Glickfeld et al., 2013), although it remains uncertain whether PV and SST neurons produce quantitatively similar effects on pyramidal cell output (Atallah et al., 2012; Lee et al., 2012; Wilson et al., 2012; Seybold et al., 2015). In contrast, VIP neurons primarily inhibit other inhibitory neurons (Pfeffer et al., 2013; Pi et al., 2013) and increase the gain of pyramidal cell sensory responses (Pi et al., 2013; Fu et al., 2014).

Comparatively little is known about how different sources of inhibition influence sensory perception. Perturbing PV neurons has produced conflicting results: some studies show that PV stimulation improves perceptual performance (Lee et al., 2012; Aizenberg et al., 2015), while others report impaired performance (Glickfeld et al., 2013; Guo et al., 2014). In these studies, optogenetic stimulation was always delivered before, during, and after the target stimulus, leaving uncertainty about how dynamic inhibitory signals contribute to the perception of specific sensory events. Moreover, how the activity of SST or VIP neurons augments perception remains largely unexplored. Determining the contributions of different inhibitory cell classes is central to understanding the circuits and computations in sensory cortex that underlie perception.

Here, we expressed the excitatory opsin Channelrhodopsin-2 (ChR2; Nagel et al., 2003) in either PV, SST, or VIP interneurons in mouse visual cortex (V1). The mice were highly trained, such that we could precisely measure their behavioral thresholds for detecting brief increments in visual contrast. We optogenetically potentiated V1 interneuron activity synchronously with visual stimulus presentation to determine how each interneuron class influenced task performance. Our results demonstrate that different inhibitory neurons make distinct contributions to contrast perception.

## Materials and Methods

### Mouse strains

All animal procedures followed NIH guidelines and were approved by the Institutional Animal Care and Use Committee of the University of Chicago. Mouse lines were obtained from The Jackson Laboratory. Data come from PV-Cre mice (PV; five mice, three female; Jax stock #017320; Hippenmeyer et al., 2005), SST-Cre mice (SST; three mice, one female; Jax stock #013044; Taniguchi et al., 2011), and VIP-Cre mice (VIP; three mice, one female, Jax stock #010908; Taniguchi et al., 2011). These Cre-mouse lines restrict expression of excitatory opsins to the desired class with high (>95%) specificity (Pfeffer et al., 2013). Experimental animals were heterozygous for Cre recombinase in the cell type of interest (outbred by crossing homozygous Cre-expressing strains with wild type BALB/c mice, Jax stock #000651). Mice were singly housed on a reverse light/dark cycle with ad libitum access to food. Mice were water scheduled throughout the experiments, except for periods around surgeries.

### Cranial window implant

Each mouse (three to five months old) was implanted with a headpost and cranial window to give stable optical access for photostimulation during behavior (Goldey et al., 2014). Animals were anesthetized with ketamine (40 mg/kg, i.p.), xylazine (2 mg/kg, i.p.), and isoflurane (1.2–2%, in 100% O_2_). Using aseptic technique, a headpost was secured using adhesive cement (C&B Metabond, Parkell) and a 3-mm craniotomy was made over the left cerebral hemisphere (centered 3.0 mm lateral and 0.5 mm anterior to lambda) to implant a glass window (0.8-mm thickness; Tower Optical).

### Intrinsic autofluorescence imaging

We located V1 by measuring changes in the intrinsic autofluorescence signal using epifluorescence imaging (Andermann et al., 2011). Autofluorescence produced by blue excitation (470 ± 40 nm, Chroma) was collected using a green long-pass filter (500-nm cutoff) and a 1.0× air objective (Zeiss; StereoDiscovery V8 microscope; ∼0.11 NA). Fluorescence was captured with a CCD camera (AxioCam MRm, Zeiss; 460 × 344 pixels; 4 × 3 mm field of view). For retinotopic mapping; we presented full contrast drifting Gabor stimuli (10° SD; 0.1 cycles/degree; 30°/s) for 10 s followed by 6 s of mean luminance. The response to the visual stimulus was computed as the fractional change in fluorescence during the stimulus presentation compared to the average of the last 4 s of the preceding blank.

### Viral injections and ChR2 stimulation

ChR2 injections were targeted to the monocular region of V1 based on each animal’s retinotopic map (25° azimuth, ±15° elevation). For injection, mice were anesthetized (isoflurane, 1–1.5%) and the cranial window was removed using aseptic technique. We used a volume injection system (World Precision Instruments) to inject 200 nl of AAV9-Flex-ChR2-tdTomato (∼10^11^ viral particles; Penn Vector Core) 300 μm below the pial surface. The virus was injected at a rate of 40 nl/min through a glass capillary attached to a syringe (Hamilton). Following the injection, a new cranial window was sealed in place. Several weeks after injection, we localized the area of ChR2 expression using tdTomato fluorescence. We fit a Gaussian to ChR2 fluorescence to map the area of expression. The area of ChR2 expression was similar across the three genotypes (PV, SD range 0.5–1.5 mm; SST, SD range 0.4–1.2 mm; VIP, SD range 0.8–1.1 mm). We attached an optical fiber (400-μm diameter; 0.48 nA; Doric Lenses) within 500 μm of the cranial window (i.e., within 1.3 mm of the cortex). We delivered 455-nm light from an LED (Thorlabs) through the fiber. Power calibrations were based on light at the entrance to the cannula. Optogenetic stimulation began no earlier than six weeks after injection. We prevented optogenetic stimuli from cueing the animal to respond by shielding the fiber implant with blackout fabric (Thorlabs) secured to the headpost using a custom mount.

### Behavioral tasks

Animals were water scheduled and trained to respond to changes in a visual display using a lever (Histed et al., 2012). Animals were first trained to respond to the appearance of a full-contrast Gabor stimulus on uniform background with the same average luminance. The Gabor stimulus (SD 6.75°, 0.1 cycles/deg, odd-symmetric) appeared for 100 ms, and its contrast varied randomly from trial to trial across a range that spanned behavioral threshold. Stimuli for each animal were positioned at a location that corresponded to the V1 representation expressing ChR2 (25° azimuth, ±15° elevation). Mice initiated trials by depressing the lever. After a random delay (400–3000 ms), the Gabor appeared for 100 ms, and the mouse had to release the lever within a brief response period beginning 100 ms after stimulus onset (max: 900 ms). Early releases and misses resulted in a timeout before the start of the next trial. Stimulus presentation, behavioral control and data collection and analysis were done using custom software based on MWorks (http://mworks-project.org), MATLAB (The MathWorks, Inc.), and Python.

Optogenetic stimulation did not begin until animals worked reliably for hundreds of trials each day and contrast detection thresholds were stable across days. This typically required approximately two months of training. During optogenetic experiments, we activated ChR2-expressing inhibitory neurons on a randomly selected half of the trials by delivering light through the optical fiber positioned over V1. During preliminary sessions (typically one to two), the stimulation power was adjusted to produce an approximately two-fold change in detection threshold (data from these testing sessions was excluded from the primary analyses). Once selected, the stimulation power was fixed throughout testing. To align the opsin illumination with visually evoked spiking in V1, the onset of illumination was delayed by 35 ms relative to the appearance of the visual stimulus on the monitor.

### Histology

Mice were perfused with 10% pH neutral-buffered formalin (Millipore Sigma Inc.), after which the brain was removed and submerged in fixative for 24 h. The brain was subsequently rinsed with PBS, placed in a 30% sucrose PBS solution until it sank. Brains were sectioned at 40 μm on a freezing microtome, mounted and cover slipped. tdTomato expression was visualized with 561-nm excitation using a Caliber ID RS-G4 Confocal Microscope with a 10× objective (Olympus; 0.3 NA).

### Data analysis

Detection thresholds were determined using trials in which the subject either responded correctly (hit) or failed to respond (miss; Histed et al., 2012). Performance data were first corrected for false alarms by finding the probability of observing a false alarm in each 100-ms trial bin. We then subtracted a randomly selected fraction of correct trials from each contrast level, balanced for trials with and without optogenetic stimulation, based on this false alarm rate function. This correction was typically small (median hits removed 7.0%; range 2.3–12.2% for 129 sessions from 11 mice). Corrected performance data were then fit with a Weibull cumulative distribution function using non-linear least squares and variance weighting of each mean. The two psychometric functions (with and without ChR2 stimulation) were fit simultaneously using four parameters: individual thresholds (α_unstimulated_, α_stimulated_), a common lapse rate (γ), and a common slope (β) such that:

$$\text{Proportion correct}=\left(1-\gamma \right)\times \left({1-e}^{{-\left(\frac{contrast}{\alpha }\right)}^{\beta }}\right)$$

A small fraction of sessions (19/129) were significantly better fit by the addition of a second slope parameter specific to ChR2 stimulation trials (*F* test for number of parameters). Sessions fit with five parameters were distributed across genotypes (PV: 10%; SST: 18%; VIP: 16% of total sessions) and produced similar effects of stimulation on psychometric detection thresholds (median threshold change (visual + optogenetic threshold/visual threshold) with four parameters = 1.54; median threshold change with five parameters = 1.45; *p* = 0.35; Wilcoxon rank-sum test). Threshold confidence intervals were estimated using a bootstrap (1000 repetitions, *p* < 0.05, one-tailed).

To assess how activating different interneuron classes influenced task performance, the data were fit to three different models, which were compared using log-likelihood ratios, with confidence intervals estimated using a bootstrap procedure (1000 repetitions, *p* < 0.05, one-tailed). For comparing models, performance data were not corrected for false alarms as was done previously so that we could better assess whether ChR2 stimulation produced a change in the probability that the animal would respond independent of the visual stimulus. All three models had the same number of parameters. Trials without ChR2 stimulation were fit with a threshold (α), lapse rate (γ), slope (β), and false alarm rate (δ) such that:

$$\text{Proportion correct unstimulated}=\left(\left(1-\gamma -\delta \right)\times \left({1-e}^{-{\left(\frac{contrast}{\alpha }\right)}^{\beta }}\right)\right)+\delta$$

The parameters were shared for performance data in the presence of ChR2 stimulation, with an added term (σ) that augmented the psychometric function differently depending on the model being tested. We first tested a contrast gain model in which ChR2 stimulation causes a multiplicative change in the contrast of the stimulus.

$$\text{Proportion correct contrast gain}=\left(\left(1-\gamma -\delta \right)\times \left({1-e}^{-{\left(\frac{contrast}{\alpha \times \sigma }\right)}^{\beta }}\right)\right)+\delta$$

where σ < 1 corresponds to a perceptual enhancement, and σ > 1 corresponds to a perceptual impairment.

We also tested a contrast addition model, in which ChR2 stimulation adds a fixed increment or decrement to the contrast of the stimulus.

$$\text{Proportion correct contrast addition}=\left(\left(1-\gamma -\delta \right)\times \left({1-e}^{-{\left(\frac{contrast+\sigma }{\alpha }\right)}^{\beta }}\right)\right)+\delta$$

Where σ was negative, we constrained the fit such that the proportion correct was δ (false alarm rate) whenever contrast + σ was negative, under the assumption that optogenetic stimulation should not impact spontaneous lever releases.

Lastly, we tested a model in which ChR2 stimulation augments the probability of lever release (response probability) independent of the visual stimulus. This model takes different forms depending on whether the response probability is decreased:

$$\text{Proportion correct response probability}=\left(\left(\left(1-\gamma -\delta \right)\times \left({1-e}^{-{\left(\frac{contrast}{\alpha }\right)}^{\beta }}\right)\right)+\delta \right)\sigma$$

or increased:

$$\text{Proportion correct response probability}=\left(\left(\left(\left(1-\gamma -\delta \right)\times \left({1-e}^{-{\left(\frac{contrast}{\alpha }\right)}^{\beta }}\right)\right)+\delta \right)+\sigma \right)-\left(\left(\left(1-\gamma -\delta \right)\times \left({1-e}^{-{\left(\frac{contrast}{\alpha }\right)}^{\beta }}\right)\right)+\delta \right)\sigma$$

In the case of ChR2 detection in the absence of visual stimuli (VIP-Cre mice), we fit a three-parameter psychometric function (α, β, γ) after correcting for false alarms as above. False alarm rates in ChR2 detection experiments were comparable to experiments with both visual and optogenetic stimuli (percentage of trials removed; median 6.8%; range 5.8–12.8%; *n* = 17 sessions from two mice).

## Results

Our primary goal was to determine how different classes of inhibitory neurons contribute to visual contrast perception. We used transgenic mouse lines that express Cre-recombinase selectively in one of the three major genetic subclasses of inhibitory neurons in mouse cerebral cortex: PV, SST, and VIP (Hippenmeyer et al., 2005; Taniguchi et al., 2011). Prior work has shown that these mouse strains allow selective targeting of excitatory opsins to the interneuron class of interest with >95% specificity (Pfeffer et al., 2013).

**Figure 1.**
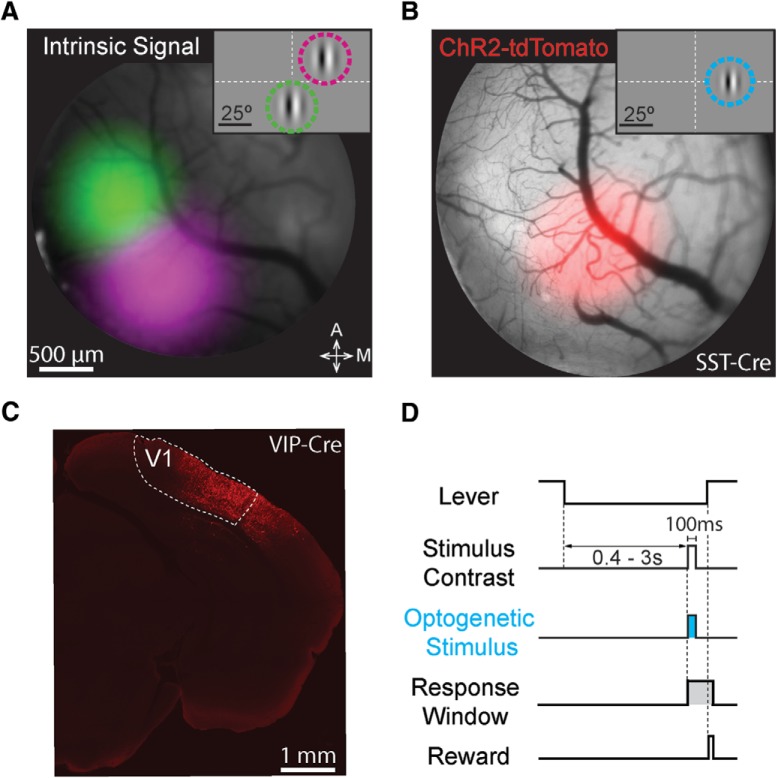
Targeting ChR2 to retinotopically defined areas of visual cortex. ***A***, Pseudo-colored intrinsic autofluorescence responses to visual stimuli presented in two locations in a SST-Cre mouse. Magenta and green features represent 2D-Gaussian fits of responses to stimuli at visual field locations depicted in the inset (green: 0° azimuth, –20° elevation; magenta: 25° azimuth, +20° elevation; Gabor SD = 10°). Dashed lines represent horizontal and vertical meridians. A: anterior; M: medial. ***B***, ChR2-tdTomato fluorescence (2D-Gaussian fit) from the same cortical region shown in ***A***. The retinotopic location corresponding to maximal expression was used in all behavioral sessions (shown in inset; 25° azimuth, 0° elevation; Gabor SD = 6.75°). Conventions as in ***A***. ***C***, Representative confocal image of ChR2-tdTomato expression in the visual cortex (V1) of a VIP-Cre mouse. ***D***, Trial schematic of the contrast increment detection task. Following the intertrial interval, a trial begins when the mouse depresses the lever. A visual stimulus could appear from 400–3000 ms following trial onset. The mouse had to release the lever within 700–900 ms after stimulus onset to receive reward. On a randomly selected half of the trials, ChR2-expressing interneurons were illuminated with blue light for 100 ms concurrent with the visual stimulus.

Each mouse was surgically implanted with a headpost and a cranial window to give stable optical access to V1 (Goldey et al., 2014). Following implantation, we mapped V1 using intrinsic signal imaging (Fig. 1*A*) to guide injections of a Cre-dependent virus containing ChR2-tdTomato (Fig. 1*B*,*C*; Nagel et al., 2003). Mice were trained to do a contrast increment detection task while head fixed. In this task (Fig. 1*D*), the mouse faced a video display filled with a mid-level gray. To start a trial, it depressed and held a lever through a randomly varying delay period (400–3000 ms) after which a stimulus appeared briefly (100 ms). The mouse had to release the lever within 700–900 ms to receive a reward.

We used static, vertically-oriented, monochromatic, odd-symmetric Gabor patches (25° azimuth, ±15° elevation, 6.75° SD; 0.1 cycles/degree). Visual stimuli were presented at a visual field location corresponding to the V1 representation with strongest ChR2 expression (Fig. 1*B*). We varied the visual stimulus contrast between trials to measure psychophysical detection threshold. An optical fiber was attached to the headpost to ensure optogenetic stimulation would affect neuronal activity in a consistent location each day. Optogenetic stimulation was delivered concurrently with the visual stimulus (100-ms duration beginning at visual stimulus onset; Fig. 1*D*).

### Visual detection is impaired by PV or SST neuron activation and enhanced by VIP neuron activation

**Figure 2.**
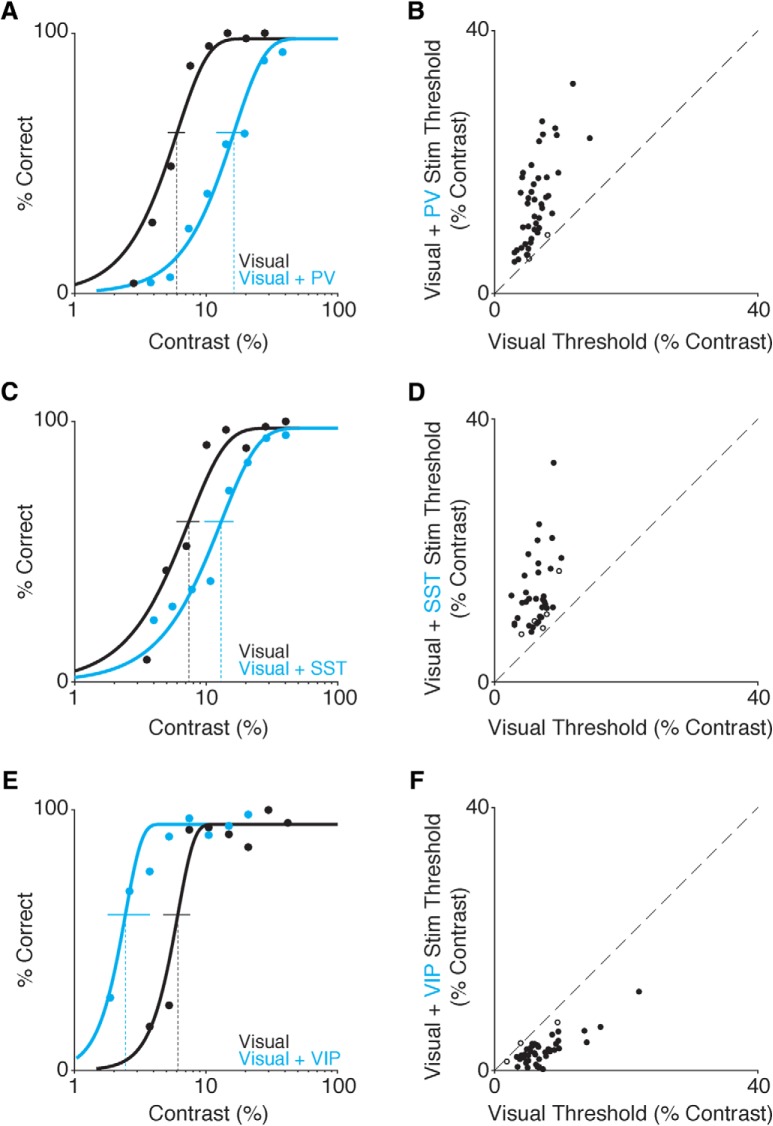
PV and SST stimulation impairs, while VIP stimulation improves, contrast increment detection. ***A***, Representative PV mouse behavioral performance from a single contrast increment session. Data are false-alarm corrected performance for trials with (blue) and without (black) activation of PV interneurons as stimulus contrast is varied. Curves are best-fitting Weibull functions that were used to determine detection thresholds (dotted vertical lines) and 95% confidence intervals (solid horizontal lines). ***B***, Summary of PV stimulation effects. Circles depict the contrast increment detection thresholds from individual sessions with (*y*-axis) and without (*x*-axis) PV neuron stimulation (five mice, 47 sessions). Filled circles indicate a significant shift in threshold (44/47; bootstrap). ***C***, Representative behavioral performance from a single session in a SST mouse. Same format as ***A***. ***D***, Summary of SST stimulation effects (three mice, 39 sessions; significant threshold difference in 34/39 sessions). Same format as ***B***. ***E***, Representative single session behavioral performance from a VIP mouse. Conventions as in A and ***C***. ***F***, Summary of VIP stimulation effects (three mice 43 sessions; significant threshold difference in 40/43 sessions). Same format as ***B***, ***D***.

We began optogenetic stimulation experiments after contrast increment detection thresholds were stable. The optogenetic stimulus power was held constant throughout a session at a level chosen ensure that mice could still reliably report high contrasts. Such settings produced an ∼2× change in threshold and roughly corresponded to the relative proportions of each interneuron subtype (average power, PV: 0.85 mW; SST: 0.85 mW; VIP: 2.0 mW). Powers were selected during one or two preliminary screening sessions before experimental data collection that were not included in the main analyses. We measured the effects of optogenetic stimulation by fitting Weibull functions to behavioral data collected with and without optogenetic stimulation (Fig. 2). To isolate the effects of interneuron activation on detection threshold, the fitting procedure was constrained to a common lapse rate and slope with independent thresholds for trials with and without optogenetic stimulation (for equations, see Materials and Methods). Contrast detection thresholds measured without optogenetic perturbation were very similar across genotypes (median threshold contrast, PV: 6.0%; SST: 6.6%; VIP: 6.5%; H(2) = 0.61, *p* = 0.74; Kruskal–Wallis test).

Optogenetic stimulation of PV neurons impaired contrast increment detection, resulting in rightward shift of psychometric functions (Fig. 2*A*). Across all PV sessions (five mice, 47 sessions), contrast increment detection thresholds were significantly higher with PV stimulation, whether considering medians (median 13.6% vs 6.0%, *p* < 10^−8^; Wilcoxon signed-rank test; Fig. 2*B*) or the average ratio of thresholds with and without optogenetic stimulation (average ratio 2.2, SEM 0.1; range 1.0–4.3). Performance was never enhanced by PV stimulation and bootstrap analysis (see Materials and Methods) showed that PV stimulation significantly impaired detection on most of the individual daily sessions (44/47; Fig. 2*B*, filled symbols). It should be noted, however, that the magnitude of the change depends on arbitrary experimental factors such as the power of the optogenetic illumination, the level of ChR2 expression and the precise positioning of the optic fiber. Indeed, during preliminary testing, we found that the change in performance produced by optogenetic perturbations scaled approximately linearly with illumination power (data not shown).

Optogenetic stimulation of SST interneurons similarly impaired contrast increment detection, shifting psychometric functions to the right (Fig. 2*C*). Across all SST stimulation sessions (three mice, 39 sessions), contrast increment detection thresholds were significantly greater when the visual stimulus was paired with SST activation (median 12.0% vs 6.6%, *p* < 10^−7^; Wilcoxon signed-rank test; average ratio 2.2, SEM 0.1; range 1.1–5.1; Fig. 2*D*). SST stimulation significantly impaired detection on most individual daily sessions (34/39). Overall, enhancing either PV or SST interneuron mediated inhibition similarly impaired the detection of contrast increments.

In stark contrast to PV and SST perturbations, optogenetic stimulation of VIP neurons enhanced contrast increment detection, shifting psychometric functions to the left (Fig. 2*E*). Across all VIP sessions (three mice, 43 sessions), contrast increment detection thresholds were significantly lower in the presence of VIP stimulation (median 2.8% vs 6.5%, *p* < 10^−7^; Wilcoxon signed-rank test; Fig. 2*F*). VIP activation also produced about a two-fold change in thresholds, but unlike PV and SST stimulation, it was a halving of detection thresholds (0.4, SEM 0.03; range 0.02–1.0). VIP stimulation significantly improved detection on most individual daily sessions (40/43). Thus, VIP interneurons make a contribution to visual perception that is opposite to that made by PV and SST interneurons.

### The change in performance likely results from a change in perceptual sensitivity

**Figure 3.**
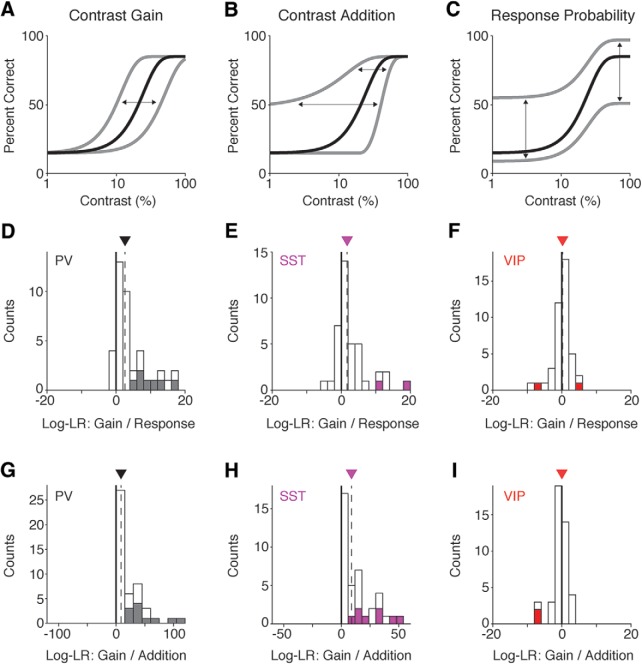
Optogenetic effects on detection performance are well described by changes in perception rather than response probability. ***A–C***, Hypothetical examples of how optogenetic stimulation of interneurons could impact detection performance. Black line = performance on trials without stimulation. Gray line = impairment/enhancement in performance resulting from interneuron stimulation. Arrows indicate direction and magnitude of shifts in detection performance resulting from optogenetic stimulation. Lapse and false alarm rates have been exaggerated to highlight differences in model predictions. ***A***, If stimulation impacts sensory evidence by causing a multiplicative change in contrast, the psychometric function should shift along the horizontal axis. ***B***, If stimulation adds a fixed increment or decrement to the contrast of the stimulus, the psychometric function should shift primarily at low contrasts. ***C***, If stimulation affects performance by changing the response probability, the psychometric function should shift above the lapse rate or below the false alarm rate. ***D***, Distribution of log-likelihood ratios from individual sessions comparing the contrast gain model to the response probability model in PV mice. ***E***, Same as in ***D*** but for SST mice. ***F***, Same as in ***D*** but for VIP mice. ***G***, Distribution of log-likelihood ratios from individual sessions comparing the contrast gain model to the contrast addition model in PV mice. ***H***, Same as in ***G*** but for SST mice. ***I***, Same as in ***G*** but for VIP mice. Thick black lines mark 0, where neither model had a higher likelihood. Downward pointing triangles and dashed lines denote the median of each distribution. Filled boxes are sessions for which the log-likelihood of one model is significantly better than the other model (*p* < 0.05, bootstrap). Log-LR = log-likelihood ratio. Note the differences in scale for ***G–I***.

Enhancing the activity of cortical interneurons augments sensory-evoked spiking in pyramidal output cells (Atallah et al., 2012; Lee et al., 2012; Wilson et al., 2012; Fu et al., 2014). If our optogenetic perturbations impacted perceptual processing (e.g., the sensory evidence encoded by the cortical population), the change in detection performance should be well described by a term that augments a perceptual component of the psychometric function. For example, optogenetic stimulation could increase the effective stimulus contrast multiplicatively (contrast gain; Fig. 3*A*) or additively (contrast addition; Fig. 3*B*). Alternatively, optogenetic stimulation might not affect sensory encoding but instead alter the probability that the mouse will release the lever (response probability), perhaps by affecting ongoing motor/premotor processes or the cognitive state of the animal (Fig. 3*C*).

We tested these competing possibilities by fitting the behavioral performance from each session to all three models (contrast gain, contrast addition, response probability) and computing the log-likelihood ratio for pairs of models (see Materials and Methods). For PV and SST mice, we found that the contrast gain model was superior to a model in which optogenetic stimulation reduced the response probability. For PV stimulation, the contrast gain model was favored over the lever release model in 91% (43/47) of sessions (median log-likelihood ratio for gain/response 2.6, IQR 1.8–12.6; Fig. 3*D*). The contrast gain model was significantly more likely than the response model in 30% (14/47) of all sessions. SST stimulation produced similar results. 77% of sessions (30/39) favored the contrast gain over the response model (median log-likelihood ratio for gain/response 1.7, IQR 0.3–4.3; Fig. 3*E*) and the log-likelihood ratio significantly favored the contrast gain model in 5% (2/39) sessions, while the response model was never significantly favored. In contrast, the effects of VIP stimulation did not strongly favor either the contrast gain or response model. The contrast gain model was more likely in 58% (25/43) of sessions (median log-likelihood ratio for gain/response 0.20, IQR –1.1–1.5; Fig. 3*F*). Either model was found to be significantly more likely in only a single session (both 1/43). Taken together, this suggests that optogenetically activating PV or SST interneurons impaired detection performance primarily by affecting sensory processing rather than interfering with ongoing motor or cognitive processes. As described below, VIP stimulation influences performance in different and more complex ways.

There remains considerable debate about whether PV or SST inhibition acts in a subtractive or divisive manner (Atallah et al., 2012; Lee et al., 2012; Wilson et al., 2012; Seybold et al., 2015). Given that activation of PV or SST neurons appeared to influence performance by changing the effective contrast of the stimulus, we wanted to see whether this was due to an additive or multiplicative effect. We calculated the log-likelihood ratios for the contrast gain versus contrast addition models obtained from each session. Contrast gain was strongly favored for both PV and SST mice. For PV mice, the log-likelihood ratio favored the contrast gain model over the contrast addition model in 100% (47/47) of sessions (median log-likelihood ratio for gain/addition 8.6, IQR 2.3–34.8; Fig. 3*G*). Furthermore, the contrast gain model was significantly more likely in 23% (11/47) of sessions. For SST mice, the log-likelihood ratio favored the contrast gain model in 100% (39/39) of sessions (median log-likelihood ratio for gain/addition 8.9, IQR 1.4–20.8; Fig. 3*H*) and the contrast gain model had a significantly higher likelihood in 20% (8/39) of all sessions. A similar analysis for VIP stimulation did not strongly favor either the contrast gain or contrast addition model. Contrast gain was favored in 42% (18/43) of sessions (median log-likelihood ratio for gain/addition 0.00, IQR –0.52–0.24; Fig. 3*I*). No sessions (0/43) had a significant log-likelihood ratio in favor of the contrast gain model, while 5% (2/43) of sessions significantly favored the contrast addition model. These data indicate that the effects of PV or SST stimulation likely impair performance through reducing contrast gain rather than by reducing the apparent contrast of the visual stimulus by a fixed amount.

### Mice can reliably report isolated VIP interneuron stimulation

**Figure 4.**
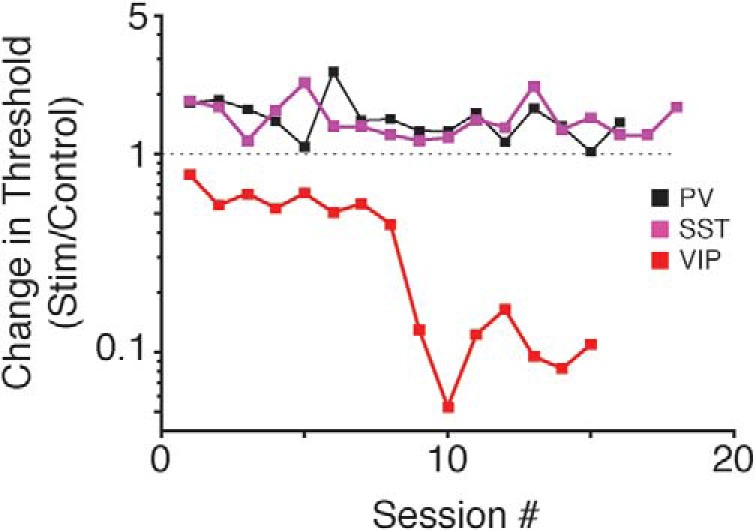
Stability of optogenetic effects on contrast detection performance. Data from three representative mice showing that extended training does little to mitigate the impairment induced by PV (black) or SST (magenta) stimulation, while VIP (red) performance improves further with training. Squares depict the ratio of thresholds from visual + optogenetic stimulus trials relative to visual only trials for each session. Values greater than one represent a performance impairment and values less than one represent performance improvement.

To examine whether animals could detect V1 interneuron activation in the absence of a visual stimulus, we tracked optogenetically induced changes in threshold across daily sessions. Figure 4 plots optogenetically induced threshold changes across days for representative mice of each genotype. Stimulation of PV (black) or SST (magenta) interneurons always impaired perception (threshold changes greater than one). These animals were representative of all PV and SST mice (*n* = 8 mice; 86 sessions): PV or SST stimulation never improved performance. The slopes of lines fit to the example data from the PV and SST mice were not significantly different from zero (PV: *t*_(14)_ = –1.78, *p* = 0.1; SST: *t*_(16)_ = –0.77, *p* = 0.45; *F* test for linear regression), suggesting that the impairment produced by optogenetic stimulation of PV or SST neurons was stable across sessions. No PV or SST mouse showed a significant reduction in the threshold impairment during our experiments. Thus, mice did not learn to detect or discount PV or SST activation.

**Figure 5.**
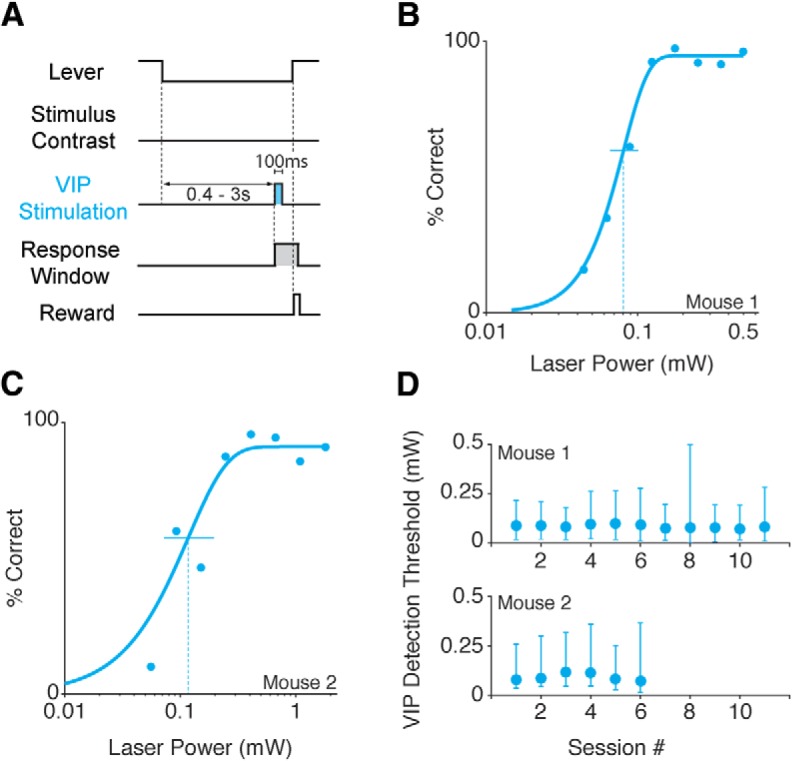
Mice can reliably report optogenetic stimulation of VIP neurons in the absence of a visual stimulus. ***A***, Trial schematic of the optogenetic stimulation detection task. The visual display contained a blank gray screen throughout the session. At a random time 400–3000 ms after trial onset a 100-ms square pulse of blue light was delivered to ChR2-expressing VIP neurons, and the mouse was required to release the lever within the reaction time window to receive reward. ***B***, Representative psychometric performance of optogenetic stimulation detection in a VIP mouse. Dots represent false-alarm corrected performance as the intensity of VIP stimulation varied across trials. The curve is the best-fitting Weibull function, which was used to determine detection threshold (dotted vertical line) and 95% confidence interval (solid horizontal line). ***C***, Same as in ***B*** for a second VIP mouse. ***D***, Thresholds are stable across sessions. Top, VIP detection thresholds (95% CI) for 11 sessions from the mouse shown in ***B***. Bottom, VIP detection thresholds (95% CI) for six sessions from the mouse shown in ***C***.

In contrast to the stable impairments due to PV or SST activation, thresholds associated with VIP stimulation continued to improve over extended stimulation sessions (Fig. 4, red line). Thresholds on visual-only trials were similar early and late in training (median 6.5% vs 6.4%; *p* = 0.66; Wilcoxon rank sum test). We attribute the change to the mice directly detecting a percept created by the activation of VIP neurons (i.e., a phosphene), and using it to guide their behavioral responses. To examine this directly, we tested whether mice could be trained to respond to VIP neuron stimulation in the absence of any natural visual stimulus. By progressively lowering the contrast of the visual stimulus, animals were conditioned to respond 100-ms periods of VIP interneuron activation in isolation (Fig. 5*A*). During each session, the video display was set to a mid-level gray and optogenetic stimulation was presented for 100 ms at a randomly selected time during each trial. We varied the intensity of the isolated optogenetic stimulus and measured the detection threshold for two mice (Fig. 5*B*,*C*). Threshold estimates were similar between the subjects (Fig. 5*B*,*C*) and relatively consistent across sessions (mouse 1: mean threshold 0.09 mW, range 0.07–0.11 mW; mouse 2: mean threshold 0.10 mW, range 0.08–0.14 mW; Fig. 5*D*). Thus, the activity of VIP neurons produces changes in neuronal activity in sensory cortices that are perceptible and these changes can be used to guide behavioral responses.

## Discussion

We have shown that optogenetic activation of different interneuron classes produces distinct effects on visual contrast perception. The ability of mice to detect increments in visual contrast was impaired by stimulation of either PV or SST interneurons, but enhanced by stimulation of VIP cells (Fig. 2). The effects of PV and SST stimulation were more closely linked to alterations in sensory evidence rather than changes in response probability (Fig. 3). Finally, the perceptual impairments produced by PV or SST stimulation were stable over several weeks, whereas mice could reliably detect optogenetic stimulation of VIP neurons in the absence of any visual stimulation (Figs. 4, 5).

We used transgenic mice to restrict expression of optogenetic actuators to genetically defined cell classes. However, even within such a class, there exists substantial heterogeneity (Markram et al., 2004; Rudy et al., 2011). SST neurons exhibit layer dependent differences in activity (Muñoz et al., 2017) and diverse electrophysiological and morphologic properties within a cortical layer (Nigro et al., 2018). Similar differences have been reported for VIP interneurons (Prönneke et al., 2015). Thus, genetic labels likely do not capture the full diversity of interneuron effects on contrast perception. Nevertheless, our results provide new insights into how distinct sources of inhibition influence visual perception and highlight the types of neuronal signals that produce perceptible patterns of activity in downstream circuits.

### Comparisons with previous work

While multiple labs have investigated how PV, SST, and VIP stimulation affects visual representations (Adesnik et al., 2012; Atallah et al., 2012; Lee et al., 2012; Wilson et al., 2012; Cottam et al., 2013; Pi et al., 2013; Fu et al., 2014), relatively few studies have examined their role in perception, and with differing results. In all prior work on PV neurons, cells were stimulated before, during and after the onset of the target stimulus. However, dynamic inhibitory signals play a role in the perception of specific sensory events. We sought to study the effects of dynamic inhibitory responses that occur coincident with stimuli by restricting optogenetic perturbations to the brief stimulus epoch. Our data indicate that activating distinct subtypes of interneurons concurrently with the onset of the visual stimulus can affect perception. Our behavioral results are supported by earlier work examining the role of PV neurons in visual and somatosensory cortex (Glickfeld et al., 2013; Guo et al., 2014). These studies included neurophysiological recordings that showed reduced stimulus-evoked responses at the light intensities that affected behavior, suggesting the impairments in perceptual performance resulted from reduced spike output from sensory cortices.

A different study found that optogenetically enhanced PV activity in mouse V1 improved orientation discrimination (Lee et al., 2012). The authors attributed this improvement to a sharpening of V1 tuning curves by PV stimulation. However, orientation discrimination thresholds were not measured. Instead the discriminability of specific, fixed orientation differences was assessed using a *d’* measure, which can improve significantly if perturbations cause the animal to withhold responses on a fraction of all trials. Moreover, the largest perceptual improvements were observed for large changes (90°) in orientation, where sharpening of tuning curves should have little effect on discrimination. Subsequent work showed that optogenetic stimulation of PV neurons impaired orientation discrimination thresholds (Glickfeld et al., 2013), suggesting that when mice are perceptually limited on orientation discrimination, PV-mediated effects in V1 do not improve orientation discrimination. This is supported by work in barrel cortex, where optogenetic activation of PV neurons impairs object location discrimination (Guo et al., 2014).

Another study found that optogenetically activating PV neurons in auditory cortex improved the ability of mice to detect a target stimulus presented on a background tone (Aizenberg et al., 2015). Interestingly, stimulus-evoked neuronal responses (relative to baseline firing rates) were enhanced by PV stimulation and this enhancement was correlated with the perceptual improvement across animals (Aizenberg et al., 2015). While potentiation of stimulus-evoked responses by enhanced inhibition is counterintuitive, simulated networks with high-gain excitatory units and strong stabilizing inhibitory feedback (Inhibition Stabilized Networks, or ISNs; Tsodyks et al., 1997; Sadeh et al., 2017) can exhibit this behavior following perturbations of the inhibitory population. Experimental evidence suggests that both visual and auditory cortices can operate in an ISN-like regime (Ozeki et al., 2009; Kato et al., 2017; but see Atallah et al., 2012; Moore et al., 2018). The findings of Aizenberg and colleagues might differ from our results because the more sustained photostimulation used in their study moved the cortical network to a different response regime. Future experiments could reconcile these differences.

Discrepancies such as these highlight challenges in using circuit perturbations to understand inhibitory neuron function. Strong optogenetic activation or suppression can produce new equilibrium states in cortex, not unlike those observed in ablation studies, that complicate interpretations of inhibitory neuron function (Sadeh et al., 2017). Furthermore, interdependent network interactions might mask the true nature of inhibition (Seybold et al., 2015). The choice of opsin also plays a critical role. Optogenetically exciting or inhibiting interneurons can produce asymmetrical effects on sensory responses (Phillips and Hasenstaub, 2016). Several opsins support optical inhibition (Wiegert et al., 2017) but can produce unintended effects on cellular activity or neurotransmitter release (Raimondo et al., 2012; Mahn et al., 2016), whereas different excitatory opsins produce a large range of photocurrents even with equivalent levels of expression (Klapoetke et al., 2014).

### Relating changes in neuronal output to behavior

There have been conflicting reports as to whether the activity of PV and SST neurons modulates cortical responses to visual stimuli in an additive or multiplicative fashion (Atallah et al., 2012; Lee et al., 2012; Wilson et al., 2012). We found that the behavioral impairments produced by PV or SST activation were more like a multiplicative change rather than an additive change in contrast. Our data are consistent with a recent report indicating that optogenetic activation of either PV or SST neurons reduces neuronal responses primarily multiplicatively (Phillips and Hasenstaub, 2016). Our results are consistent with expectations based on the way each interneuron type influences pyramidal cell spiking. A straightforward strategy for detecting contrast increments is to integrate spikes from sensory neurons and respond when the count exceeds a criterion value (Geisler, 2011). Pyramidal cell responses to sensory stimuli are increased by VIP activation (Pi et al., 2013), and decreased by PV or SST activation (Atallah et al., 2012; Cottam et al., 2013). Thus, the altering stimulus-evoked spiking in V1 could accelerate (VIP) or impede (PV/SST) progress toward a detection criterion. Indeed, we previously showed that detection of V1 activity is well described by linear integration of inputs over short (100 ms) intervals (Histed and Maunsell, 2014). Thus, the elimination of spikes by PV or SST stimulation and the addition of spikes resulting from VIP stimulation is a straightforward framework for explaining the observed changes in detection performance.

VIP neurons primarily suppress the activity of SST neurons (Pfeffer et al., 2013), with the net effect of amplifying the responses of pyramidal neurons to visual stimuli (Fu et al., 2014). The improvement that occurred with extended training shows that the effects of VIP stimulation were not stationary. A process like perceptual learning has been described in monkeys responding to electrical stimulation of V1 (Ni and Maunsell 2010), and a similar process might have occurred in our mice during VIP stimulation. What the current experiments cannot address is whether the nature of the underlying effect of VIP activation also changed over time. It is possible that VIP activation acted only to increase the apparent contrast of the visual stimulus. Alternatively, VIP stimulation might have produced a percept that was obviously distinct from, but synchronous with, the visual stimulus. In either case, improvements in behavioral threshold over time would arise from the robustness of the VIP signal growing with training.

It is also possible that VIP stimulation initially increased the apparent contrast of the visual stimulus, but eventually transitioned to an independently detectable percept. This last scenario might explain why neither the perceptual nor response models were superior for VIP mice. In most sessions, VIP stimulation might have produced a combination of enhanced stimulus representation (perceptual model) and neuronal activity that could generate responses independently from the visual stimulus (response model). Given that none of the three models we tested for VIP mice was clearly superior, we cannot conclusively distinguish between these competing possibilities. Future studies will be needed to resolve the nature of VIP-mediated effects on visual perception.

Given that optogenetic activation of V1 pyramidal neurons is perceptible (Histed and Maunsell, 2014) and VIP stimulation increases pyramidal cell spiking (Pi et al., 2013), VIP stimulation is likely to become detectable by virtue of activating V1 pyramidal neurons that project to other structures. Despite comparable amounts of training, optogenetic stimulation always impaired perception in PV and SST mice. Given the well-established effects of PV and SST activity on pyramidal cell output, our data raise the intriguing possibility that decrements in V1 activity do not produce perceptible patterns of activity in downstream structures. It has previously been suggested that retinal OFF pathways exists to convert stimulus decrements into an excitatory signal (Schiller, 1992). Opposed, rectified signaling channels can provide greater sensitivity and reduced latencies for spiking networks. It is possible that brain structures downstream of V1 decode changes in stimulus intensity based solely on the activity of neurons that increase their firing in response to those changes. This possibility might be addressed by detailed experiments examining correlations between population spiking patterns and particular perceptual outcomes.

## Synthesis

Reviewing Editor: Tatiana Pasternak, University of Rochester Decisions are customarily a result of the Reviewing Editor and the peer reviewers coming together and discussing their recommendations until a consensus is reached. When revisions are invited, a fact-based synthesis statement explaining their decision and outlining what is needed to prepare a revision will be listed below. The following reviewer(s) agreed to reveal their identity: Anirvan Nandy, Adam Snyder.

Both reviewers found this study of the role of different subtypes of inhibitory interneurons on perceptual thresholds well-done and the results largely convincing. However, they raised a number of issues that must be addressed before the paper can be considered for publication in eNeuro. These are outlined below.

1. Please address potential implications of the temporal asynchrony in the delivery of visual and optogenetic stimulation

2. Please comment on the summation fields of different interneuron classes and whether the difference in these field could explain observed differential effects

3. Please explain how the basis for selecting the power of optogenic stimulation. How was the criterion of a two-fold change in threshold was chosen?

4. Reviewer 2 identified problems with model fitting data analysis and its validation. Please clarify your model and your approach to its validation. Do you agree with this reviewer that an alternative approach involving data likelihood would be better?

5. Please address the possibility that the VIP stimulation effect could be explained by a "redundant target" effect.

6. Reviewer 2 identified a few statements in the Discussion that may need rephrasing, including the point about suppression of sensory responses by optogenetic stimulation and about PV and SST mice not detecting optogenetic stimulation.

REVIEWER 1

The authors investigate the contributions of different classes of interneurons to sensory perception. They do this by optogenetic stimulation of PV+, SST+ & VIP+ interneurons in mouse V1 while the animals are engaged in a contrast change detection task. They report that while PV & SST simulation impairs detection of contrast, VIP stimulation enhances contrast detection. The results are consistent with the canonical architecture of the PV/SST/VIP micro-circuit with the principal cells.

Overall, the study is well conducted and the paper is clearly written. I had a few issues that I would like the authors to address.

(A) The logic behind synchronous delivery of visual and optogenetic stimulus was not clear. The experiment as conducted implies that the optogenetic stimulation enhanced the activity of the targeted interneuron sub-type ~30-40ms before the visual information reached V1; similarly, the optogenetic enhancement of the targeted sub-type was removed ~30-40ms before the tail end of the visual information. In effect, the two kinds of stimulations (visual and optogenetic) were asynchronous in the cortex. Can the authors comment on the implications of this asynchrony? How would the results change if the stimulations were concurrent in the cortex?

(B) Can the authors comment on the summation fields of the different interneuron types? Might some of the results hinge on the extent of the viral expression in the different mouse lines (this was not quantified in the study; although it appears from Fig 1A-C that the extents might have been different) and the summation fields of the targeted interneuron subtypes?

(C) In the 3rd paragraph of p6, the authors state: "The optogenetic stimulus power was held constant throughout a session at a level chosen to produce ~2x change in threshold ...". It was not clear how this level was chosen without doing the experiment in the first place. Was this done in preliminary experiments that the authors do not report?

REVIEWER 2

ADVANCES IN THE FIELD

There is great interest to understand how distinct cell classes contribute to perceptual processes. This is difficult to ascertain by observation alone because causality is elusive in that case. The key manipulation would be to selectively activate one cell class at a time, which these authors have done using optogenetic methods. Therefore I think the work would be of interest to readers of eNeuro and it advances the field.

STATISTICS

In my comments to the authors I describe how the "Additive" model assumes impossible scenarios (i.e., performance better than 100% correct) and therefore is not an appropriate hypothesis as stated in the manuscript. Also the method for quantifying model fitness is suboptimal, which I explain further in the comments to authors.

COMMENTS TO THE AUTHORS

The authors optogeneticially stimulated different genetic subtypes of inhibitory neurons in visual cortex of mice performing a contrast increment detection task. They report that stimulation of parvalbumin-expressing and somatostatin-expressing neurons increases contrast increment detection thresholds, whereas stimulation neurons expressing vasoactive intestinal peptide led to decreased perceptual thresholds. The work seems like a good addition to the growing literature regarding the role of inhibitory neurons in the neural circuitry underlying modulation of sensory processing. My only real concern regards the analysis in which competing models are fit to the data. Lesser concerns are related to the details of reporting, as well as matters for further discussion.

Concerns:

1) I'm not entirely on board with the model-fitting analysis. First, it would be helpful to specify the models explicitly with mathematical formulae, pointing out the free parameters, etc. Second, the scenario depicted in the bottom panel of Figure 3a is not possible, because the animals are performing at ceiling levels for high contrast values in the unstimulated condition, and the percent correct cannot exceed 100%. A related potential problem is that performance cannot be worse than 0%; therefore, if the manipulation is not sufficiently strong to lift behavior off of the floor at low contrasts, then I'm not sure the picture would look much different from the one shown at the top of Figure 3a. In short, I think that the additive model fits the data less well because it does not take into account the effect of bounded performance levels and can therefore not be consistent with the observed data.

2) The authors compare the quality of the two models by computing correlation coefficients between the observed data and the fitted model values. The authors do not mention whether this was done with a held-out cross-validation set of data, or rather if the same data were used to both fit the model parameters and also evaluate the quality of the fit. Cross-validation should be used.

3) Moreover, correlation is a poor measure of model fit because it is a normalized measure and could therefore assign a high correlation between two data sets that are dramatically different scales. In this case, the authors would expect not only the shape of the psychometric function to be explained by the model, but also the exact performance values. A more straightforward method evaluating the quality of the models would be to use the data likelihood. That is, each fitted psychometric function defines a probability distribution function for Bernoulli trials at each contrast value. The probability of observing a cross-validation set of data under each model can therefore be easily calculated, and the relative likelihood of the two models compared. This approach would have the advantage of having interpretable units. For example, if the relative likelihood of the perceptual model relative to the additive model equals 2, then this means that the perceptual model is twice as likely as the additive model, etc.

4) When correcting for false alarm rate, did the authors ensure the number of trials removed was matched for each condition? If it was just "random" then there's some chance a spurious effect was introduced.

5) Does fitting a model with two slope terms favor the "perceptual" over the "additive" interpretation?

6) The authors argue that VIP stimulation improved contrast increment detection. However, an alternative explanation is a redundant targets effect. That is, since the two types of stimulation are mutually informative, detecting either is sufficient. To best resolve the ambiguity another experiment could be performed in which task-irrelevant VIP stimulation is time-independent of the occurrence of a visual target. The next best thing would be an experiment in which task-imperative VIP-only stimulation is interlaced with task-imperative visual-only stimulation as well as the redundant target. Then it could be tested whether P(detectRedundant) > 1-((1-P(detectVIP))*(1-P(detectVisual))). As it stands, the authors cannot rule out redundant target effects and this should be discussed.

7) In the discussion the authors state, "our data indicate that suppressing sensory responses concurrently with the onset of the visual stimulus can affect perception." However, the data presented do not in themselves show suppression of sensory responses, so this claim should be rephrased.

8) In the discussion the authors state, "despite comparable amounts of training, PV or SST mice never learned to detect optogenetic stimulation of these interneuron classes." I agree that this is probably likely, but the authors did not test this hypothesis directly, as they did with the VIP mice, so this should probably also be rephrased. It could have been the case, for example, that the animals' deficits would have been even more severe, but for an ability to detect the optogenetic stimulation.
